# A dynamical approach to generate chaos in a micromechanical resonator

**DOI:** 10.1038/s41378-021-00241-6

**Published:** 2021-02-19

**Authors:** Martial Defoort, Libor Rufer, Laurent Fesquet, Skandar Basrour

**Affiliations:** Univ. Grenoble Alpes, CNRS, Grenoble INP, TIMA, 38000 Grenoble, France

**Keywords:** Engineering, Physics

## Abstract

Chaotic systems, presenting complex and nonreproducible dynamics, may be found in nature, from the interaction between planets to the evolution of weather, but can also be tailored using current technologies for advanced signal processing. However, the realization of chaotic signal generators remains challenging due to the involved dynamics of the underlying physics. In this paper, we experimentally and numerically present a disruptive approach to generate a chaotic signal from a micromechanical resonator. This technique overcomes the long-established complexity of controlling the buckling in micro/nanomechanical structures by modulating either the amplitude or the frequency of the driving force applied to the resonator in the nonlinear regime. The experimental characteristic parameters of the chaotic regime, namely, the Poincaré sections and Lyapunov exponents, are directly comparable to simulations for different configurations. These results confirm that this dynamical approach is transposable to any kind of micro/nanomechanical resonator, from accelerometers to microphones. We demonstrate a direct application exploiting the mixing properties of the chaotic regime by transforming an off-the-shelf microdiaphragm into a true random number generator conforming to the National Institute of Standards and Technology specifications. The versatility of this original method opens new paths to combine the unique properties of chaos with the exceptional sensitivity of microstructures, leading to emergent microsystems.

## Introduction

Micro- and nanoelectromechanical systems (M/NEMSs) have become essential building blocks for the development of modern technologies due to their small size, low cost, and compatibility with microelectronics, with various applications such as sensors^1^, actuators^2^, or clocks^3^. In addition to their exceptional properties, optimized for high-end products, these mechanical structures are also remarkable tools for fundamental physics, both for classical^4^ and quantum^5^ investigations. This duality has triggered studies aiming to exploit the singular properties of nonlinear dynamics for direct applications in micro/nanostructures by taking advantage of the synchronization phenomenon to enhance MEMS accelerometers^6^, using internal resonances to reduce frequency drifts for timing purposes^7^ or operating microcantilevers in the nonlinear regime for mass sensing applications^8^.

Among these various nonlinear phenomena that improve M/NEMS performance, the chaotic regime features some of the most singular properties, addressing the complex needs of true random number generators^9^, secured communications^10^, or sensing applications^11,12^, but has yet to be implemented with a convenient, generic approach. Chaotic behaviors describe a large variety of involved interactions, from characterizing the evolution of cosmic entities^13^ to interpreting the unpredictability of weather^14^. Inherently complex, chaotic signals share some properties with noise, having a broad frequency spectrum and an apparent irreproducibility due to their exponential sensitivity to the initial conditions. This unique property yielded various works in electrical circuits, which were among the first physical chaotic systems tailored^15^, and on the generation of chaos in lasers for optical telecommunications applications^16^.

Due to their versatility and their large, tunable nonlinearity^17^, M/NEMSs are prime candidates for chaos studies and applications. The Duffing nonlinearity, defined as a cubic stiffness, is the most common source of chaos in M/NEMSs, which is achieved by buckling the mechanical structure using specific geometries^18^, materials^19^, or configurations^20^. The system enters a bistable configuration between the buckled up and down states, defined by a double-well potential. By driving this device with a sufficiently large force, the structure may switch between the two states, and using the appropriate driving frequency, the system experiences a chaotic regime. However, buckling micro/nanostructures at will is demanding, and while some buckled MEMS devices have experimentally demonstrated a chaotic regime^21–24^, research on the topic is mostly performed only analytically or numerically^25–27^. Among the experimental issues, the realization of the buckling states usually requires high voltages of tens to hundreds of volts^21,22,28^, and the large amplitudes involved outrange the linear regime of the commonly used transduction schemes^25^. In addition, the buckling property of the structure itself greatly reduces the range of applications of the generated chaos, and only a few works suggest nonbuckling alternatives^24,29^, which remain difficult to accomplish or investigate.

In this paper, we experimentally demonstrate the realization of a chaotic system based on the modulation of the driving signal^30^ and present a direct application. The only two requirements are to (1) obtain the Duffing regime, present in most micro/nanoresonators for a sufficiently large drive, and (2) perform either amplitude modulation (AM) or frequency modulation (FM) on the driving force. This technique is readily applicable in most current devices, as neither the fabrication process nor specific geometries are required. Considering the latest micro/nanotechnological advances, for which each application results in optimized designs, this implementation of chaos opens a path toward combining the intrinsic sensitivity of micro/nanodevices with the various properties of chaos, leading to new, emergent systems.

In the following paragraphs, we present the working principle of this chaotic regime. We characterize its properties through bifurcation diagrams, Poincaré sections and Lyapunov exponents, mapping the range of the chaotic regime in the AM and FM configurations. We compare these results with simulations involving no adjusting parameters. Finally, we demonstrate that this system may be used as a true random number generator conforming to the National Institute of Standards and Technology (NIST) specifications.

## Results

### Working principle and device

**Fig. 1:**
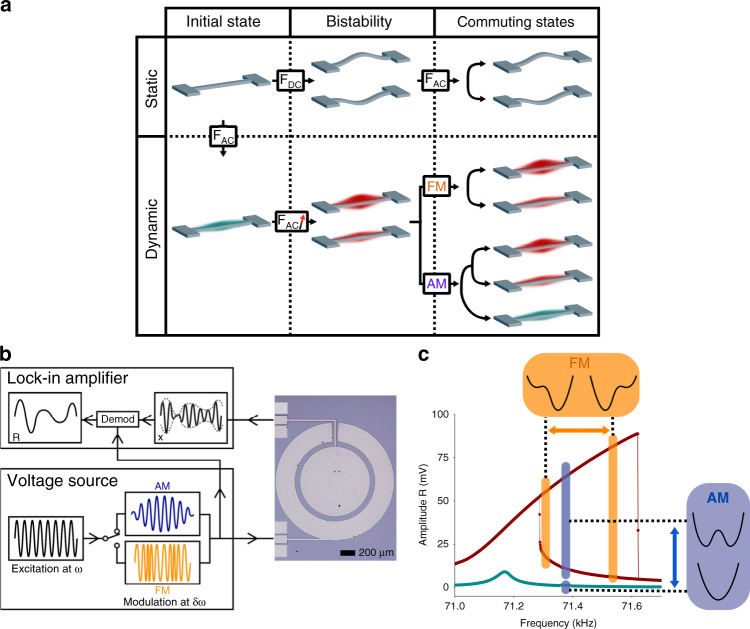
Dynamic bistability for the generation of a chaotic regime in a nonlinear resonator. **a** Comparison between static and dynamic bistability for chaos generation in a nonlinear resonator, here represented by a doubly clamped beam. In the static case, a resonator reaches bistability through buckling by applying a static force F_DC_, while in the dynamic case, the alternative driving force F_AC_ is increased to enter the Duffing regime. By modulating the driving signal, the system evolves between the different states available, which results in a chaotic regime for an appropriate set of parameters. **b** Experimental setup. The MEMS (top view photography) is driven by a voltage source that provides the AM or FM configurations. The LIA demodulates the displacement x of the MEMS at the frequency of the source output to retrieve the amplitude R. **c** Amplitude response of the MEMS in the linear and Duffing regimes (dark cyan and dark red at 10 mV and 100 mV drive, respectively). The schematics qualitatively illustrate the evolution of the potentials involved for both the amplitude and frequency modulations (AM and FM) as a guide for the reader and are not meant for quantitative comparisons. In particular, the driving amplitudes and frequencies are far from the set of parameters used in the rest of the paper

The generation of a chaotic regime is performed mostly with multistable systems. In the mechanical domain, this property is found in buckled structures, which are built by applying a constraint to the device, leading to *static* bistability (top panel of Fig. 1a). These states are defined by a static double-well potential, each well describing the buckled up and down states^21^. A chaotic regime emerges from the complex evolution between the buckled states due to an additional driving force.

However, most of these structures may instead be driven close to the resonance with a strong enough force to reveal their nonlinear behavior within the Duffing regime. In this regime, the resonator vibrates either with a large or a low amplitude^17^ (bottom panel of Fig. 1a), leading to a *dynamic* bistability described by a dynamic double-well potential. For the buckled structure, the Duffing regime may be used as the starting point to generate chaos by modulating the driving signal to commute between the different available states^30^. However, in contrast with the static case, this dynamic bistability relies only on the intrinsic properties present in most M/NEMSs, making this chaotic regime achievable for off-the-shelf devices. In this paper, we present the case of a typical circular diaphragm.

The proof-of-concept structure was fabricated using a standard multiuser MEMS process provided by Memscap under the brand name PiezoMUMPs. This process is CMOS compatible and allows multilayered structures of dimensions complying with our specifications^31^. The MEMS is a silicon-on-insulator-based circular diaphragm with a radius of 750 µm and a thickness of 10 µm (Fig. 1b and Supplementary Information (SI), Fig. S1) placed under vacuum at room temperature. The first flexural mode of the structure is actuated with a voltage source and detected with a lock-in amplifier (LIA) assisted by a current amplifier using the outer and inner electrodes coupled to the resonator, comprised of a piezoelectric 500 nm thick AlN layer. At the first order, this resonator is described by a 1D model for which its dynamics corresponds to the canonical equation:

1 $$\mathop {x}\limits^{..} + {\Delta}\omega \dot x + \omega _0^2x + \frac{{8\;\omega _0}}{3}\alpha \;x^3 = \frac{F}{m}\cos \left( {\omega \;t} \right)$$

where ${\Delta}{\it{\upomega }} = 2\pi \,{\Delta}{\it{f}}$, $\omega _0 = 2\pi f_0$, α and m are the bandwidth, angular natural resonance frequency, Duffing nonlinear coefficient and mass of the resonator, respectively, with $Q = \frac{{\it{f}}}{{{\Delta}{\it{f}}}}$ being the quality factor. With the present device, we fit a natural resonance frequency f_0_ = 71.2 kHz, a bandwidth Δf = 50 Hz leading to a quality factor Q = 1420, and a nonlinear coefficient α = 2π × 54 kHz/V^2^ (see SI, Fig. S2). The structure is driven at an angular frequency ω = 2πf by a force F. In our case, this force results from a voltage difference applied to the piezoelectric layers of the diaphragm. Note that the chaotic regime presented in this paper does not depend on the transduction mechanism and remains compatible with capacitive or optical techniques. To characterize the vibrating signal of the mechanical structure, we perform a rotating frame approximation at the driving frequency (see SI, note 1), giving the following first order:

2.a $$\dot R = - \frac{{{\Delta}\omega }}{2}R - \frac{F}{{2\;m\;\omega }}\sin \varphi$$

2.b $$\dot \varphi = \omega _0 - \omega + \alpha \;R^2 - \frac{F}{{2\;m\;\omega \;R}}\cos \varphi$$

where *R* is the demodulated amplitude—the envelope of the signal, and φ is its phase delay with respect to the driving force. The nonlinear term α *R*^2^ shifts the resonance frequency, which results in a hysteresis, at the essence of the Duffing nonlinearity (Fig. 1c), where the MEMS evolves either with a large or a low amplitude for the same driving frequency. The state of the resonator in the hysteresis depends on the history of the system, and the amplitude of vibration may switch between the high and the low level in the presence of a perturbation^32,33^. At this point, (2) describes only a bistable potential: an additional parameter is necessary to create an evolution of this dynamical system and possibly generate chaos.

The first configuration we describe performs an amplitude modulation on the driving force, namely, $F \to F\frac{{1 + \cos \delta \omega \;t}}{2}$, with $\delta \omega = 2\pi \delta f$ being the angular modulation frequency of the signal, which is directly transposable to (2) (see SI, note 1). With a modulation depth of 100%, the force experienced by the system oscillates from 0 to *F* at the rate *δω*. If *F* is large enough to open the hysteresis of the Duffing resonator, the system oscillates between a linear regime with a single-well potential and a nonlinear regime with a double-well potential (Fig. 1c, blue areas).

The second configuration consists of a frequency modulation where the driving phase becomes $\omega \;t \to \omega \;t + \sin \left( {\delta \omega \;t} \right)$, which has a modulation index of 1. In (2), it follows that $\omega \to \omega + \delta \omega \;\cos \left( {\delta \omega \;t} \right)$ (see SI, note 1). This essentially corresponds to back and forth sweeps through the resonance such that the system may switch between the high-amplitude and low-amplitude states in the Duffing regime (Fig. 1c, orange areas).

For slow modulations ($\delta \omega \ll {\Delta}\omega$), the system may switch between its two states but with no additional exotic behavior. However, when the modulation rate becomes comparable to the inverse of the system’s time response, the resonator’s dynamics becomes more complex, and new physics may emerge. In the following section, we consider a modulation rate of three times the resonator bandwidth.

### Bifurcation diagram and Poincaré sections

To characterize the evolution of a system from the periodic to the chaotic regime, a common approach is to generate a bifurcation diagram^34^. It consists of a stroboscopic view of the amplitude of a signal, sliced at the modulation frequency *δf*, as a given parameter is swept (in our case, the driving frequency *f*). These bifurcation diagrams enable us to characterize the route to chaos of the system, which usually consists of an increasing number of harmonics in the signal, until reaching the chaotic regime with a broad frequency spectrum.

**Fig. 2:**
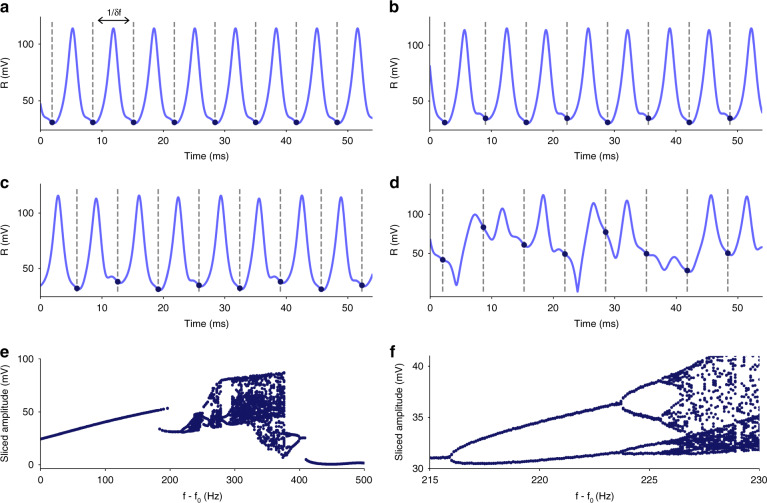
Experimental bifurcation diagram in the AM configuration for a driving signal of 0.5 V and a modulation of 151 Hz. To characterize the route to chaos, experimental data are sliced (dark blue dots) with a period of 1/*δf* (dashed lines) for different driving frequencies. Typical mechanical responses are presented (from **a** to **d**) for detuning frequencies *f*−*f*_0_ of 215, 220, 225, 340 Hz, respectively, which highlight the presence of 1, 2, and 4 harmonics in the signal, until the chaotic regime is reached. The projection of the sliced data for each driving frequency forms a bifurcation diagram (**e**). Looking closer at its evolution before entering chaos (**f**), we observe the period-doubling route to chaos with period-doubling bifurcations up to $\frac{{\delta f}}{8}$

We generate a bifurcation diagram as a function of the driving frequency close to the natural resonance frequency (Fig. 2). First, the modulation of the driving signal is accurately reproduced by the structure, with low distortion (Fig. 2a). However, as the driving frequency progresses through the hysteresis, the Duffing nonlinearity alters the mechanical response to the modulated driving signal, leading to a more complex yet periodic amplitude of vibration with higher harmonics (Fig. 2b–c) until reaching a chaotic regime (Fig. 2d). A closer look at the bifurcation diagram before entering the chaotic regime (Fig. 2f) reveals a period-doubling route to chaos, where the subharmonics present in the signal increase by a factor of two at each bifurcation point. In the AM configuration, we measured period-doubling bifurcations up to $\frac{{\delta f}}{8}$. This route to chaos displays universal properties such as the constants of Feigenbaum^35^, ultimately enabling us to predict the threshold value after which the chaotic regime appears.

**Fig. 3:**
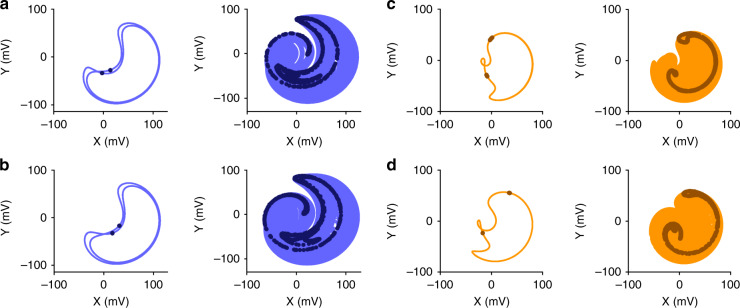
Phase space and Poincaré sections of the linear and chaotic responses of the resonator. The experimental phase spaces of the AM and FM configurations (**a** and **c**) are compared with the numerical results (**b** and **d**, respectively). In the linear regime (left section of each panel), the periodic signal is in a limit cycle configuration and repeats itself endlessly (light-colored lines). The stroboscopic analysis reveals two periods in this example (dark-colored points). In the chaotic regime (right section of each panel), the signal is no longer periodic and fills up the phase space. The Poincaré section is composed of an infinite number of nonoverlapping points that exhibit a specific signature. This signature is reproducible even in the chaotic regime, as demonstrated by the numerical analysis performed with no adjustable parameters. These signals were generated with a modulation rate of 151 Hz with different (drive, frequency detuning) sets of parameters. In the AM configuration, we used (0.5 V, 220 Hz) in the periodic regime and (0.5 V, 340 Hz) in the chaotic regime. In the FM case, we used (0.15 V, 155 Hz) in the periodic regime and (0.15 V, 175 Hz) in the chaotic regime

This stroboscopic view is also at the basis of the Poincaré sections, which extract order from the apparent noisy structure of a chaotic signal^36^. Similar to bifurcation diagrams, a sufficiently long dataset is sliced at the modulation frequency with an arbitrary initial time. The sliced data are gathered on a graph presenting the phase space of the system, commonly shown as the displacement versus velocity. In the present scenario, the variables are the in-phase (*X* = *R* cos *φ*) and the out-of-phase (*Y* = *R* sin *φ*) components of (2). For a given combination of fixed parameters, the global shape of a Poincaré section has a specific, reproducible signature. For a periodic signal, the signature represents a finite set of overlapping points, depending on the periodicity. For a chaotic signal, it forms a pattern of nonoverlapping points (Fig. 3). The longer the dataset is, the more precise the pattern of the chaotic Poincaré section becomes. Exploiting (2) with either amplitude or frequency modulation, the Poincaré sections of the chaotic signals are directly simulated with the measured experimental parameters of the system, quantitatively reproducing the experimental data.

While raw chaotic signals appear random, the specific signatures of the Poincaré section analysis leave clues, partially revealing the nature of the chaotic system. Being able to numerically reproduce their shapes gives a lever to build more complex chaos with potential applications in cryptography.

### Lyapunov exponents and TRNG

One of the main aspects of a chaotic signal is its unpredictability and irreproducibility. These properties are related to the system’s sensitivity to the initial conditions, often categorized within three scenarios. In a damped oscillator, any initial mismatch between two similar measurements progressively shrinks over time. In a driven resonator, an initial phase delay mismatch remains constant over time. In a chaotic resonator, any mismatch increases over time. This property is characterized by the Lyapunov exponent *λ*, following $\delta z\left( t \right) = \delta z_0\;e^{\lambda \;t}$, with *δz* being the distance between two initially close trajectories^37^, resulting in (1) converging trajectories (*λ* < 0), (2) conservative trajectories (*λ* = 0), and (3) diverging trajectories (*λ* *>* 0). The measurement of this exponent is based on finding the neighbor states in an acquired signal. In a periodic signal, any arbitrarily picked state is reproduced after one period. Hence, there is one new neighbor state after each period. For a chaotic signal, a sufficiently long acquisition ensures the eventual identification of close states, giving access to the local maximum Lyapunov exponent (SI, Fig. S3). After an even longer measurement, enough pairs of different initial states are gathered, enabling computation of the global Lyapunov exponent^38^.

**Fig. 4:**
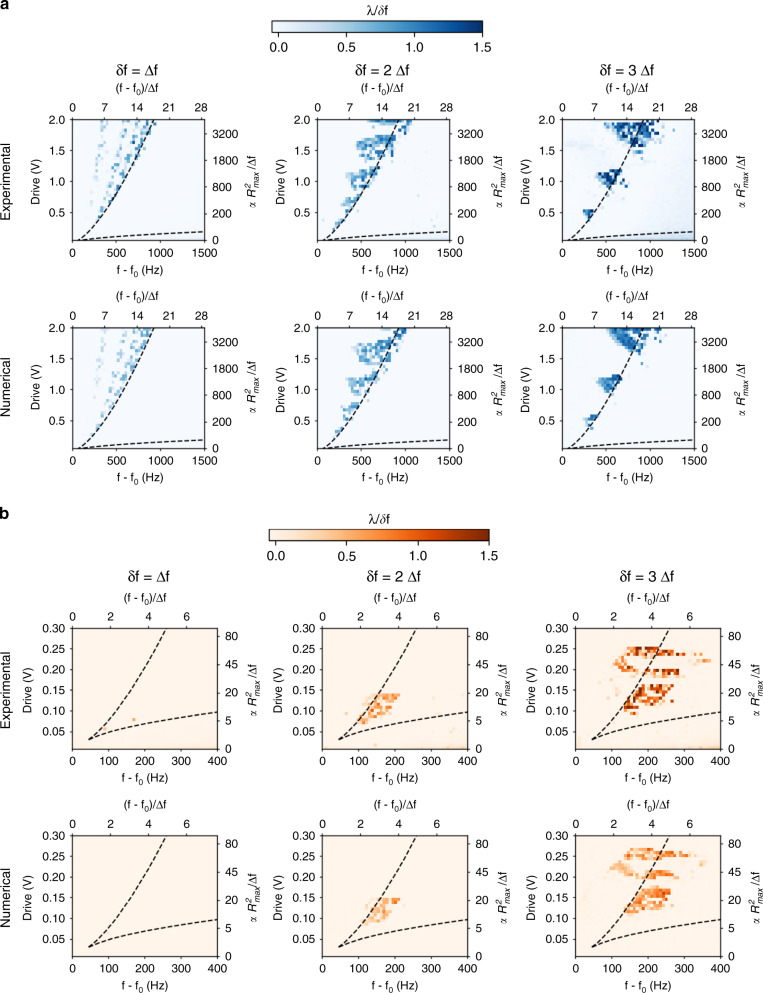
Mapping of the Lyapunov exponents in both the AM and FM configurations. A positive global Lyapunov exponent is characteristic of a chaotic regime, and we use its value to determine the chaotic range as a function of the driving frequency and the driving force for modulation rates of one, two and three times the bandwidth of the system in both the AM (**a**) and FM (**b**) configurations. Note that the Lyapunov exponents are normalized to the modulation rate and that the right/top axis are normalized to the bandwidth of the system for a more comprehensive view of the phenomenon. The experimental Lyapunov maps are compared with numerical simulations carried out using the measured experimental parameters. The black dashed lines represent the two edges of the hysteresis in the Duffing regime and therefore the bistable and monostable regions

By changing the driving force and frequency for a fixed modulation rate, we mapped the regions where the MEMS has a chaotic response through the measurement of this Lyapunov exponent both experimentally and numerically (Fig. 4). We chose three modulation rates corresponding to one, two and three times the bandwidth of the linear resonator, for which the associated maps present different trends depending on the modulation configuration. In the AM case, the chaotic region appears to be bondless, and while the presented results stop for an equivalent force of 2 V, we kept measuring chaotic signals with the same modulation rates up to 3.5 V. In the FM configuration, the chaotic regime requires a lower force for a similar modulation rate. The chaotic region also broadens with the modulation rate but is confined in terms of the force, and we could not measure any chaos above the voltages presented. In both cases, the chaotic regime follows a similar behavior as a function of the three physical parameters. The minimal driving amplitude is always above the onset of the Duffing regime, which is essential to obtain bistability. A modulation rate of one bandwidth is barely enough to measure chaos, which can be interpreted as the system being at the onset of the out-of-equilibrium state. Finally, the frequency shift necessary to obtain chaos is close to the left edge of the hysteresis (left dashed line in Fig. 4). It seems counterintuitive to observe chaos occurring outside the bistable region, in particular in the AM configuration. However, it is important to remember that the system is not just driven at these voltages and frequencies but is also modulated. The system will experience bistability as long as the modulation (in amplitude or frequency) crosses the bistable region, which is the case for all of the chaotic regimes probed in Fig. 4. Note that this explanation goes beyond the simplified schematics in Fig. 1c, which were meant to give only a first intuition of the phenomenon.

The difference in the force range between AM and FM can then be understood as follows. In the AM configuration, for each driving force, there will always be a detuning frequency for which the modulation will enable access to both wells. In the FM configuration, we restricted our study to the case of a modulation index of 1. Therefore, the frequency span of the modulation is fixed at a given modulation rate, while the size of the hysteresis increases with the driving force. The distance between the two wells of the hysteresis then becomes out of reach.

In both cases, the averaged Lyapunov exponent grows with the modulation rate, which indicates a smaller memory time. The experimental chaotic regions defined by λ are quantitatively reproduced numerically, demonstrating that simple system (2) is precise enough to encompass the behavior of the chaotic resonator despite the large variation between each modulation rate. The higher the driving amplitude and modulation rate are, the larger the frequency range of the chaotic regime becomes, enabling them to remain in the chaotic regime even in the presence of frequency drifts. Since no assumptions were made regarding the geometry or nature of the resonator, system (2) is readily applicable to any Duffing resonator—the right and top axes of Fig. 4 were normalized to ease its application (see SI, note 2). The modulation rate needed to obtain chaos is not limited to the presented results; this regime is also achievable for larger drives with a modulation rate of up to at least 30 times the bandwidth (see SI, Fig. S4).

**Fig. 5:**
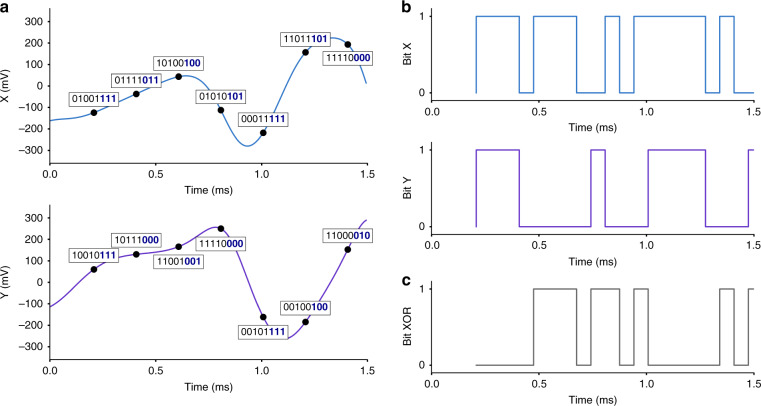
Signal processing for the TRNG application. Slicing the data with a sampling rate of 5 kHz, we convert the analog signal of both X and Y using a virtual 8-bit ADC (**a**). We then extract the three least significant bits of each measurement (bold, blue) and generate a bit sequence (**b**). We finally combine both the X and Y sequences through an XOR function to generate the final bit stream, producing a random sequence at a rate of 15 kb/s (**c**). The chaotic signal was obtained for a 3.5 V drive and a 1250 Hz frequency detuning in the AM configuration at a modulation rate of 1.5 kHz

The Lyapunov exponent describes the memory of the system. As such, the prediction of the evolution of a chaotic signal is limited by the measurement precision. This feature is well suited for true random number generation, and physical chaotic systems have already proven to pass standard randomness tests^9,39–41^ such as NIST SP 800-22^42^. By itself, chaos provides only a complex way to mix an initial state, which is deterministic and predictable and is the source of some numerical pseudorandom number generators^43^ (PRNGs). However, if the initial state is noisy, the predictability exponentially vanishes in the chaotic regime. Therefore, the combination of a stochastic seed (e.g., intrinsic thermomechanical noise or frequency fluctuations^44^) with the exponential sensitivity of chaos may turn the system into true random number generators (TRNGs). To demonstrate this application, we performed NIST tests on the output signal of our MEMS in the chaotic regime, which is converted into a binary form through a numerical analog-to-digital convertor (ADC). To add up the mixing properties of the chaos, we used a common process consisting of selecting the least significant bits (LSBs) of an 8-bit ADC digitizing the analog chaotic signal^39,40^ (Fig. 5a). The sampling rate can then be much faster than *λ*. In our case, we sampled both X and Y components of the signal at 5 kHz (Fig. 5b). Keeping solely the 3 LSBs of each measurement, we then performed an XOR function between the X and Y sequences, known to improve the randomness of a bit stream (Fig. 5c).

We finally obtained a sequence of 75 Mb divided into 75 sequences of 1 MB, processed through the NIST tests, showing that the chaotic system passes all the tests (see SI, Table 1) and delivers a random bit stream at a rate of 15 kb/s. As the chaotic system seed is noisy, a stochastic model of the entropy extraction can be obtained to prove the true system randomness. The performance of this TRNG can be tuned according to the specific needs, such as the bit rate or power consumption. Due to the very nature of this chaos (imprinted as a modulation of the amplitude of the resonator), the bit rate of this TRNG is arguably smaller than that of traditional buckled-based chaos (imprinted directly on the displacement of the resonator). In addition, the strong nonlinearity of buckled devices increases their complexity and possibly their potential for TRNG applications, at the cost of a substantially larger driving power. In contrast, the dynamical method to generate chaos is noninvasive, such that most current M/NEMS devices initially designed for specific goals such as accelerometers or gyroscopes could additionally be used for true random number generation, provided a modulation scheme and a demodulation scheme are available.

We stress that the MEMS structure used in this paper was not designed nor optimized for this chaotic regime. An improvement in chaos complexity, rate and power consumption may easily be performed through a higher Duffing nonlinearity, a higher resonance frequency and a lower bandwidth while maintaining a qualitative understanding of the system. Note that most of these systems can be obtained by moving to lower dimensions with NEMS technology.

In parallel to our work, recent studies also highlighted the potential of dynamic bistability for chaos generation through two drive tones^45^ and amplitude modulation^46^. However, in the first case, the interpretation of the chaos generation is drastically different, as the second tone is seen as a perturbation creating a libration orbit^45^, and the second approach focuses on a very specific working regime of the amplitude modulation, preventing a broad understanding of the generated chaos^46^. In contrast, our study allows us to draw a comprehensive overview of the phenomena at stake by illuminating the role of the different parameters of the system. In particular, our results point toward new opportunities and implementations impacting future technologies, which we illustrate with the generation of true random numbers, pillars of modern security and data protection.

## Discussion

Chaotic systems are known to present unique properties for cryptographic applications, which we illustrate in this paper with an experimental demonstration of an original chaotic MEMS-based TRNG. While being far from the figures of merit of optical or electronic chaotic TRNGs, having unequal bit rates and footprints to date, MEMS devices enable an in situ fine adjustment of their relevant parameters. In addition to the rate, size or power, the quality of a TRNG also lies in its capability to face malicious attacks. Adjusting directly the physical parameters of a system with feedback control could offer a lever to counteract disruptive attacks.

This affinity between chaos and secured communication reaches a capstone with chaos synchronization^16^, at the core of most research on chaotic lasers for telecommunication applications and recently also for mechanical systems^46,47^. While the optical domain benefits from a large bandwidth for unequaled data rates, micro/nanomechanical resonators are tunable over orders of magnitude with a resolution below ppm, providing a large number of cryptographic keys, essential for secured transmissions. In addition to cryptography, the capacity to build a chaotic system from current mechanical structures opens up new perspectives to study experimentally unchallenged chaos properties, especially in the field of noise filtering.

Remarkably, chaos is weakly sensitive to noise^48,49^, implying that a stochastic process will have a negligible effect on the chaotic regime of a system. Conversely, a deterministic signal coupled to a chaotic system could trigger a bifurcation from the chaotic to the periodic regime, thereby amplifying the detection of the deterministic signal. Combining both properties, a chaotic system becomes a noise-free sensor, and numerical simulations have demonstrated the detection of signals buried within more than 60 dB of noise^11^. This unmatched property lacks experimental demonstration, and M/NEMSs are prime tools for this study, being at the frontier between fundamental and applied research.

Through the chaotic regime presented in this paper, combining the noise-free property of chaos with the high sensitivity of M/NEMSs is at hand, which will not only result in emergent sensors but also help unravel the many complex features of this singular chaotic property.

In conclusion, we presented experimentally and numerically a disruptive method to generate a chaotic signal from a nonlinear MEMS structure. The only requirement for the system is to present a Duffing nonlinearity and to be able to perform either amplitude or frequency modulation on the driving signal^30^. We obtained a quantitative comparison between the experimental and numerical results, describing the chaotic complexity through the Poincaré sections and the chaotic range through the Lyapunov exponents. As a model system, a M/NEMS enables us to experimentally explore the properties of chaotic systems, for instance, making use of the control on the Lyapunov exponent by tuning the modulation rate. In addition, unlike most M/NEMS-based chaotic systems, this method does not have any geometrical or material requirements leading to buckling. This freedom enables the implementation of the chaotic regime in most resonant M/NEMSs, and we foresee that this could be the first step toward the combination of the high-precision features of M/NEMSs with the high sensitivity of chaos for sensing applications.

## Methods

### Setup

The voltage source we used is a 33500B Agilent generator, enabling both the AM and FM configurations. The measurement of the MEMS device is performed through an HF2TA Zurich Instrument current amplifier with a 10 kΩ load resistance, the output of which is then probed by an HF2LI Zurich Instrument Lock-In Amplifier. All measured voltages are in root mean square values throughout the paper. Since the bandwidth of the resonator is 50 Hz, the modulation rates of one, two and three times the bandwidth are purposely shifted by 1 Hz to avoid 50 Hz noise from the electrical lines.

### FM configuration

As suggested by the SI, note 1, the FM configuration requires demodulating the signal at the modulated frequency of the source. This is easily performed when the generator and the demodulator belong to the same instrument. Otherwise, as in our case, the demodulation frequency has to be synchronized to that of the generator. Without this procedure, both bifurcation diagrams and Poincaré sections would appear extremely noisy, even if the demodulator has a bandwidth much higher than the modulation rate.

Note that the FM is mathematically equivalent to a direct modulation of the resonance frequency *f*_0_ instead of the driving frequency *f*, which may be easier to perform depending on the device.

### Numerical simulations

The simulations were performed starting from Eq. (2) with either the AM or FM configuration using the PyDSTool Python library.

### Bifurcation diagrams and Poincaré sections

The datasets processed for the bifurcation diagrams and the Poincaré sections have to be recorded with a very high sampling rate, orders of magnitude higher than the modulation rate. A postprocessing analysis over undersampled data (even if the sampling rate is above the spectral bandwidth) results in a poor observation of the $\frac{{\delta f}}{n}$ bifurcation points and noisy Poincaré sections. In Figs. 2 and 3, we used a sampling rate more than two orders of magnitude higher than the modulation rate.

The bifurcation diagrams are obtained by slicing the data at the modulation rate, with any initial phase delay. However, depending on the variable of interest (in our case, the amplitude *R*), some phase delay places more emphasis on the spreading after each bifurcation point. In Fig. 2, we used a convenient initial phase delay between the modulation of the voltage source and the measured signal of 180° and recorded over 50 cycles of modulation.

This initial phase delay also changes the associated Poincaré sections, displaying more or less complexity. We used a phase delay of 180° in Fig. 3 to stay in line with Fig. 2. However, Poincaré sections require large datasets to reveal their specific signatures, and we recorded 1500 cycles in this case.

### Lyapunov exponents

Each Lyapunov map of Fig. 4 consists of 40 × 50 pixels, each of them representing a measurement of 200 periods of modulation. The sampling rate was between 30 and 100 times higher than the modulation rate. The Lyapunov exponent is extracted from the dataset using a Wolf algorithm^38^ with a relative initial minimal neighbor distance of 3e−3 and a relative final maximal distance of 3e−1.

### Standard method for chaos generation

A typical experimental process to generate chaos starts by selecting a modulation rate of a few bandwidths of the linear resonator. Then, we apply a driving amplitude of more than an order of magnitude higher than the onset of the Duffing regime in the AM configuration (less in the FM case). Finally, we sweep in frequency close to the edge of the hysteresis (the left one for a positive Duffing coefficient, the right one for a negative Duffing coefficient) over a range equivalent to a few bandwidths of the linear resonator. The authors recommend using the normalized parameter used in Fig. 4 as a starting point by looking at the top and right axis and Supplementary Note 2.

## Supplementary information

Supplementary Information

## Data Availability

The data used in this work are available directly from the corresponding authors upon reasonable request.
