# Supplementary material for: A dynamical approach to generate chaos in a micromechanical resonator
Source: Microsyst Nanoeng. 2021 Feb 19;7:17. doi: 10.1038/s41378-021-00241-6 (PMC8433204; doi:10.1038/s41378-021-00241-6)
Supplement: Supplementary file 1 — Supplementary Information [file 41378_2021_241_MOESM1_ESM.docx]

**A dynamical approach to generate chaos in a micromechanical resonator**

**Supplementary Information**

| 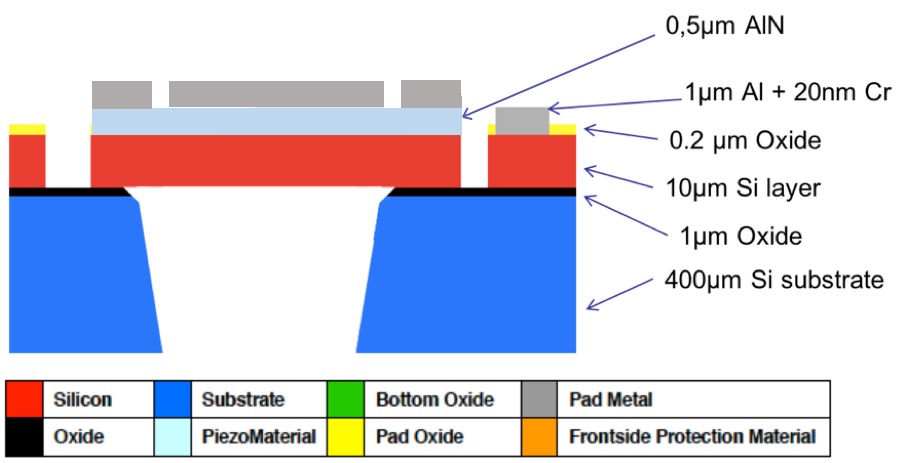 |
| --- |
| **Figure S1. Cross-section of the MEMS structure.** A silicon-on-insulator (SoI) wafer is used as a starting substrate with a 400 µm thick Si substrate and an oxide thickness of 1 µm. The diaphragm is released from the bulk silicon substrate by trench etching through the bulk substrate using deep reactive ion etching (DRIE) comprises a 10 µm thick single-crystal silicon device layer, 0.5 µm reactively sputtered AlN layer, and 1 µm of Al with 20 nm of Cr is the adhesion layer. |

|  |
| --- |
| **Figure S2. Characterization.** All amplitude units throughout the main paper and the supplementary material are in root mean square. **a** The amplitude of vibration of the structure on resonance follows a linear trend with the driving voltage (dashed grey line) up to an amplitude of around 120 mV. This amplitude corresponds to a displacement of around 10 µm, which is the thickness of the membrane, and we attribute the nonlinear trend at large drive to the large amplitude altering the material response (full black line). **b** The Duffing nonlinearity shifts the resonance frequency up quadratically with the displacement amplitude, leading to a Duffing coefficient of 54 kHz/V^2^ (full black line). Note however that in the main text, for the chaos analysis, we mostly address the MEMS close to its natural resonance frequency $f_{0}$ (far from the nonlinear resonance frequency), where the amplitude follows a linear trend with the drive. |

|  |
| --- |
| **Figure S3. Experimental measurement of a local Lyapunov exponent.** In a chaotic regime, two initially close states s_1_ and s_2_ eventually diverge (**a**, showing a window of 5 ms in AM configuration, with a drive of 1 V, a frequency detuning of 600 Hz and a modulation rate of 151 Hz), and their rate of divergence defines the local Lyapunov exponent λ (**b**, showing the same acquisition for a larger timescale). |

|  |
| --- |
| **Figure S4. Experimental chaotic regime at large modulation rate.** Chaotic characteristics of the system for an amplitude modulation rate of 1.5 kHz, demonstrating that this dynamical chaos still works for $\delta\omega\gg\Delta\omega$. **a** Poincaré section for the AM configuration (driving voltage: 3.5 V, frequency detuning: 1250 Hz). **b** Coarse Lyapunov map. |

| \| Test \| p-Value \| Proportion \| Result \| \| --- \| --- \| --- \| --- \| \| Frequency \| 0.044425 \| 75/75 \| Pass \| \| Block Frequency \| 0.754127 \| 73/75 \| Pass \| \| Cumulative Sums \| 0.622341 \| 150/150 \| Pass \| \| Runs \| 0.099089 \| 75/75 \| Pass \| \| Longest Run \| 0.491599 \| 75/75 \| Pass \| \| Rank \| 0.666838 \| 75/75 \| Pass \| \| FFT \| 0.069925 \| 74/75 \| Pass \| \| NOT Matching \| 0.419859 \| 11008/11100 \| Pass \| \| OT Matching \| 0.009343 \| 74/75 \| Pass \| \| Universal \| 0.548605 \| 74/75 \| Pass \| \| Approx. Entropy \| 0.015444 \| 74/75 \| Pass \| \| Random Excursion \| 0.433207 \| 371/376 \| Pass \| \| Random Exc. Var. \| 0.393372 \| 839/846 \| Pass \| \| Serial \| 0.650162 \| 150/150 \| Pass \| \| Linear Excursion \| 0.256632 \| 75/75 \| Pass \| |
| --- | --- | --- | --- | --- | --- | --- | --- | --- | --- | --- | --- | --- | --- | --- | --- | --- | --- | --- | --- | --- | --- | --- | --- | --- | --- | --- | --- | --- | --- | --- | --- | --- | --- | --- | --- | --- | --- | --- | --- | --- | --- | --- | --- | --- | --- | --- | --- | --- | --- | --- | --- | --- | --- | --- | --- | --- | --- | --- | --- | --- | --- | --- | --- | --- |

**Table S1: Test of randomness with NIST SP 800-22.** The test was performed using the same set of parameters as in Fig. 5.

**Supplementary Note 1:**

**Rotating wave approximation in the normal, AM and FM configurations**

**Normal configuration:**

Starting from the master equation:

$\ddot{x}+\Delta\omega\dot{x}+{\omega_{0}}^{2} x+\frac{8 \omega_{0}}{3}\alpha x^{3} = \frac{F}{m}\cos\left( \omega t \right)$ (S1)

as defined in the main text, such that the solution $X\left( t \right)$ at first order is of the form:

$x\left( t \right)=\frac{A\left( t \right) e^{i \omega t}+{A\left( t \right)}^{*} e^{-i \omega t}}{2}$,

$A\left( t \right)=R\left( t \right) e^{i \varphi\left( t \right)}$ (S2)

with $R$ and $\varphi$ the amplitude of vibration and phase delay at the driving frequency $\omega$. It follows:

$x^{3}=\frac{A^{3} e^{i 3 \omega t}+3\left| A \right|^{2}A e^{i \omega t}+CC}{8}$,

$\dot{x}=\frac{i \omega A e^{i \omega t}+\dot{A} e^{i \omega t}+CC}{2}$,

$\ddot{x}=\frac{- \omega^{2} A e^{i \omega t} + 2 i \omega\dot{A} e^{i \omega t}+\ddot{A} e^{i \omega t}+CC}{2}$ (S3)

where $CC$ stands for Complex Conjugate. Putting all the terms back to (S1) and keeping solely the $e^{i \omega t}$ terms, we are left with:

$- \omega^{2} A + 2 i \omega\dot{A}+\ddot{A}+\Delta\omega\left( i \omega A+\dot{A} \right)+{\omega_{0}}^{2} A+2 \omega_{0} \alpha\left| A \right|^{2}A=\frac{F}{m}$ (S4)

Assuming a slow varying profile ($\omega A\gg\dot{A}$) and a high-quality factor ($\omega\gg\Delta\omega$) we obtain:

$2 i \omega\dot{A}+i \omega\Delta\omega A+\left( {\omega_{0}}^{2}- \omega^{2} \right)A+2 \omega_{0} \alpha\left| A \right|^{2}A=\frac{F}{m}$ (S5)

Separating real and imaginary parts, we obtain:

$$2 \omega\dot{R}=- \omega\Delta\omega R-\frac{F}{m}\sin\varphi,$$

$2 \omega R \dot{\varphi}=\left( {\omega_{0}}^{2}-\omega^{2} \right)R+2 \omega_{0} \alpha\left| R \right|^{2}R-\frac{F}{m}\cos\varphi$ (S6)

Finally, assuming $\omega_{0}-\omega\ll\omega$, we have ${\omega_{0}}^{2}-\omega^{2}\sim2 \omega\left( \omega_{0}-\omega\right)$, we obtain (2):

$$\dot{R}=-\frac{\Delta\omega}{2}R-\frac{F}{2 m \omega}\sin\varphi,$$

$\dot{\varphi}=\omega_{0}-\omega+\alpha\left| R \right|^{2}-\frac{F}{2 m \omega R}\cos\varphi$ (S7)

**FM Configuration:**

This configuration induces some changes, as the driving frequency itself changes:

$\ddot{x}+\Delta\omega\dot{x}+{\omega_{0}}^{2} x+\frac{8 \omega_{0}}{3}\alpha x^{3} = \frac{F}{m}\cos\left( \omega t+\sin\left( \delta\omega t \right) \right)$ (S8)

at first order, the solution $X\left( t \right)$ becomes:

$x\left( t \right)=\frac{A\left( t \right) e^{i \left[ \omega t+\sin\left( \delta\omega t \right) \right]}+{A\left( t \right)}^{*} e^{-i \left[ \omega t+\sin\left( \delta\omega t \right) \right]}}{2}$,

$A\left( t \right)=R\left( t \right) e^{i \varphi\left( t \right)}$ (S9)

It is essential to demodulate at $\omega t+\sin\left( \delta\omega t \right)$ to capture chaotic patterns such as the Poincaré sections. It follows:

$x^{3}=\frac{A^{3} e^{i 3 \left[ \omega t+\sin\left( \delta\omega t \right) \right]}+3\left| A \right|^{2}A e^{i \left[ \omega t+\sin\left( \delta\omega t \right) \right]}+CC}{8}$,

$\dot{x}=\frac{i \left[ \omega+ \delta\omega\cos\left( \delta\omega t \right) \right] A e^{i \left[ \omega t+\sin\left( \delta\omega t \right) \right]}+\dot{A} e^{i \left[ \omega t+\sin\left( \delta\omega t \right) \right]}+CC}{2}$,

$$\ddot{x}=-\frac{\left( i {\delta\omega}^{2}\sin\left( \delta\omega t \right) + \left[ \omega+ \delta\omega\cos\left( \delta\omega t \right) \right]^{2} \right) A e^{i \left[ \omega t+\sin\left( \delta\omega t \right) \right]}}{2}$$

$$+ \frac{2 i \left[ \omega+ \delta\omega\cos\left( \delta\omega t \right) \right] \dot{A} e^{i \left[ \omega t+\sin\left( \delta\omega t \right) \right]}+\ddot{A} e^{i \left[ \omega t+\sin\left( \delta\omega t \right) \right]}+CC}{2} (S10)$$

Going back to (S8) and keeping solely the $e^{i \left[ \omega t+\sin\left( \delta\omega t \right) \right]}$ terms, we are left with:

$$- \left( i {\delta\omega}^{2}\sin\left( \delta\omega t \right) + \left[ \omega+\delta\omega\cos\left( \delta\omega t \right) \right]^{2} \right) A + 2 i \left[ \omega+\delta\omega\cos\left( \delta\omega t \right) \right] \dot{A}+\ddot{A}$$

$+ \Delta\omega\left( i \left[ \omega+\delta\omega\cos\left( \delta\omega t \right) \right] A+\dot{A} \right)+{\omega_{0}}^{2} A+2 \omega_{0} \alpha\left| A \right|^{2}A=\frac{F}{m}$ (S11)

Assuming a slow varying profile ($\omega A\gg\dot{A}$) and a high-quality factor ($\omega\gg\Delta\omega$) we obtain:

$$2 i \left[ \omega+\delta\omega\cos\left( \delta\omega t \right) \right] \dot{A}+i \left( \left[ \omega+\delta\omega\cos\left( \delta\omega t \right) \right] \Delta\omega-{\delta\omega}^{2}\sin\left( \delta\omega t \right) \right) A$$

$+ \left[ {\omega_{0}}^{2}- \left[ \omega+\delta\omega\cos\left( \delta\omega t \right) \right]^{2} \right] A+2 \omega_{0} \alpha\left| A \right|^{2}A=\frac{F}{m}$ (S12)

Assuming a low modulation rate ($\omega\gg\delta\omega$, $\omega\Delta\omega\gg{\delta\omega}^{2}$), we have:

$2 i \omega\dot{A}+i \omega\Delta\omega A+ \left[ {\omega_{0}}^{2}- \omega^{2}-2 \omega\delta\omega\cos\left( \delta\omega t \right) \right] A+2 \omega_{0} \alpha\left| A \right|^{2}A=\frac{F}{m}$ (S13)

As in the normal configuration, after separating real and imaginary parts, assuming $\omega_{0}-\omega\ll\omega$ and ${\omega_{0}}^{2}-\omega^{2}-2 \omega\delta\omega\cos\left( \delta\omega t \right)\sim2 \omega\left[ \omega_{0}-\omega-\delta\omega\cos\left( \delta\omega t \right) \right]$, we obtain:

$$\dot{R}=-\frac{\Delta\omega}{2}R-\frac{F}{2 m \omega}\sin\varphi,$$

$\dot{\varphi}=\omega_{0}-\omega-\delta\omega\cos\left( \delta\omega t \right)+\alpha\left| R \right|^{2}-\frac{F}{2 m \omega R}\cos\varphi$ (S14)

**AM Configuration:**

This configuration is strictly similar to the normal one, where one can replace $F$ by $F\frac{1+\cos\left( \delta\omega t \right)}{2}$ at every step all the way from (S1) to (S7).

We start with:

$\ddot{x}+\Delta\omega\dot{x}+{\omega_{0}}^{2} x+\frac{8 \omega_{0}}{3}\alpha x^{3} = \frac{F}{m}\frac{1+\cos\left( \delta\omega t \right)}{2}\cos\left( \omega t \right)$ (S15)

and finish with:

$$\dot{R}=-\frac{\Delta\omega}{2}R-\frac{F}{2 m \omega}\frac{1+\cos\left( \delta\omega t \right)}{2}\sin\varphi,$$

$\dot{\varphi}=\omega_{0}-\omega+\alpha\left| R \right|^{2}-\frac{F}{2 m \omega R}\frac{1+\cos\left( \delta\omega t \right)}{2}\cos\varphi$ (S16)

**Supplementary Note 2:**

**Normalization of the axes in Fig. 4**

The top x-axis of Fig.4 corresponds to the frequency detuning $f-f_{0}$, and is normalized to the bandwidth of the system $\Delta f$. The right y-axis corresponds to the force driving the resonator. In the case of a Duffing resonator, which behavior depends on the Duffing nonlinearity, it is convenient to convert the driving force $F$ into the associated Duffing nonlinearity $\alpha{R_{max}}^{2}=\alpha\left( \frac{F Q}{m {\omega_{0}}^{2}} \right)^{2}$. This value corresponds to the frequency shift induced by the force in the Duffing regime. It is then normalized to the bandwidth of the system.
